# How do lifestyle factors modify the association between genetic predisposition and obesity-related phenotypes? A 4-way decomposition analysis using UK Biobank

**DOI:** 10.1186/s12916-024-03436-6

**Published:** 2024-06-10

**Authors:** Mengrong Zhang, Joey Ward, Rona J. Strawbridge, Carlos Celis-Morales, Jill P. Pell, Donald M. Lyall, Frederick K. Ho

**Affiliations:** 1 School of Health and Wellbeing, University of Glasgow, Clarice Pears Building, 90 Byers Road, Glasgow, G12 8TB UK; 2 Cardiovascular Medicine Unit, Department of Medicine Solna, Karolinska Institute, Stockholm, Sweden; 3 School of Cardiovascular and Metabolic Sciences, University of Glasgow, Glasgow, UK; 4 Human Performance Lab, Education, Physical Activity, and Health Research Unit, Universidad Católica del Maule, Talca, Chile; 5 Centro de Investigación en Medicina de Altura (CEIMA), Universidad Arturo Prat, Iquique, Chile

**Keywords:** Obesity, Central obesity, BMI-PRS, WHR-PRS, Total lifestyle factors

## Abstract

**Background:**

Obesity and central obesity are multifactorial conditions with genetic and non-genetic (lifestyle and environmental) contributions. There is incomplete understanding of whether lifestyle modifies the translation from respective genetic risks into phenotypic obesity and central obesity, and to what extent genetic predisposition to obesity and central obesity is mediated via lifestyle factors.

**Methods:**

This is a cross-sectional study of 201,466 (out of approximately 502,000) European participants from UK Biobank and tested for interactions and mediation role of lifestyle factors (diet quality; physical activity levels; total energy intake; sleep duration, and smoking and alcohol intake) between genetic risk for obesity and central obesity. BMI-PRS and WHR-PRS are exposures and obesity and central obesity are outcomes.

**Results:**

Overall, 42.8% of the association between genetic predisposition to obesity and phenotypic obesity was explained by lifestyle: 0.9% by mediation and 41.9% by effect modification. A significant difference between men and women was found in central obesity; the figures were 42.1% (association explained by lifestyle), 1.4% (by mediation), and 40.7% (by modification) in women and 69.6% (association explained by lifestyle), 3.0% (by mediation), and 66.6% (by modification) in men.

**Conclusions:**

A substantial proportion of the association between genetic predisposition to obesity/central obesity and phenotypic obesity/central obesity was explained by lifestyles. Future studies with repeated measures of obesity and lifestyle would be needed to clarify causation.

**Supplementary Information:**

The online version contains supplementary material available at 10.1186/s12916-024-03436-6.

## Background

Obesity is a common condition with a global prevalence that has nearly tripled since 1975, and the World Health Organization (WHO) considers obesity to pose a significant burden on public health [1]. It is regarded as a major risk factor for chronic disease, especially for cardiometabolic disease [2]. Whilst body mass index (BMI) is the most commonly used measure of overall adiposity, waist-hip ratio (WHR) is a better measure of central adiposity which is more related to the risk of cardiometabolic disease [3].

Obesity results from complex relationships between a wide range of genetic, lifestyle, and environmental factors, rather than a sole risk factor [4]. This means that, whilst some people have a higher genetic predisposition to adiposity, their actual level of adiposity will also be influenced by their environment and lifestyle [5], such as physical activity level and energy intake [6, 7]. This is important for public health interventions. Firstly, in contrast to genetic factors, lifestyle and environment can be modifiable. Secondly, if interactions exist then non-modifiable risk factors such as genetics may, nonetheless, be useful in targeting public health interventions at those most likely to benefit; commonly referred to as precision medicine or precision public health.

Many aspects of lifestyle, including physical activity level [8], energy intake, diet quality [9], sleep duration [10], smoking [11]and alcohol consumption [12], are known to be associated with obesity and central obesity. However, it is unclear to what extent genetic predisposition to adiposity is mediated via predisposition to unhealthy lifestyle (vertical pleiotropy) or whether it operates primarily via other mechanisms. Furthermore, studies on gene-lifestyle interactions and obesity and central obesity have been limited in number and have focused on diet [13], physical activity [14, 15], and sleep [16]. Few studies have investigated whether other lifestyle risk factors, such as alcohol consumption and smoking, modify genetic predisposition to obesity.

In this study, we conducted comprehensive analyses to investigate the extent to which lifestyle factors (physical activity level, energy intake, diet quality, sleep duration, smoking, and alcohol consumption) modify the relationship between BMI and WHR polygenic risk scores (PRS) and phenotypic obesity and central obesity. We also conduct mediation analyses to quantify the extent to which the pathway from genetic predisposition to obesity is direct (independent of lifestyle) or indirect (genes predispose to lifestyle which predisposes to obesity).

## Methods

### Study design

This cross-sectional study used baseline data from the UK Biobank, a population cohort study. From April 2006 to December 2010, 502,536 participants, who were largely between 40 and 70 years old were recruited [17]. Participants attended one of the 22 assessment centres across England, Wales, and Scotland, at which they completed a touch-screen questionnaire (including self-reported physical activity level, total energy intake, diet intake, sleep duration, smoking frequency, and alcohol consumption), underwent physical measurements, and provided biological samples, as described elsewhere [18].

The main outcome measures investigated in this study were obesity and central obesity. BMI-PRS and WHR-PRS were the exposures of interest [19]. Lifestyle factors—physical activity level, total energy intake, diet quality, frequency of alcohol consumption, and smoking status—were investigated as potential mediators and effect modifiers. Sex, age, and sociodemographic deprivation were considered potential confounders and included as covariates in the statistical models, and the WHR-PRS models were stratified by sex because of a significant statistical interaction between WHR-PRS and sex.

**Fig. 1:**
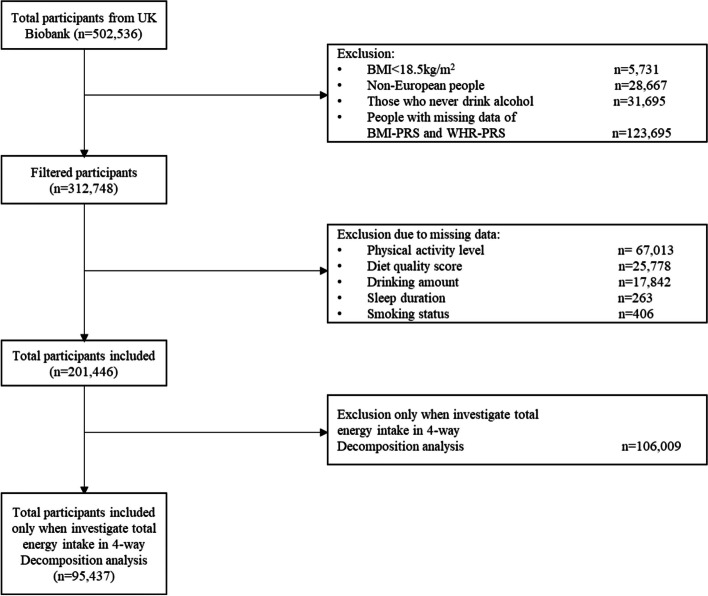
Participants flow chart

Inclusion in the study was restricted to participants who self-reported white British ethnicity to avoid heterogeneity (> 90% of the sample), and those whose BMI was ≥ 18.5 kg/m^2^ (i.e. non-underweight) to avoid non-linear associations of BMI. We excluded participants who reported never drinking alcohol because of potential confounding (e.g. stopped due to poor health), and those with missing data on BMI-PRS and WHR-PRS and/or failed genetic quality controlling. Overall, 312,748 eligible participants had genetic data available for use in this study. After excluding people who had missing data on physical activity level, diet risk score, alcohol consumption, sleep duration, and smoking status, the final study sample was *n* = 201,446. UK Biobank received ethical approval from the North-West Multi-centre Research Ethics Committee (reference: 11/NW/03820). All participants provided written, informed consent based on the principles of the Declaration of Helsinki before enrolment in the study. This project was completed using UK Biobank data application 71392 (Fig. 1).

### Outcomes and covariates

During the baseline assessment, participants’ height (Seca 202 stadiometer; Sca) and weight (BC-418 MA body composition analyzer; Tanita Corp) were measured by trained nurses [20]. Waist and hip circumference were measured using a Wessex non-stretchable sprung tape measure [21]. BMI was calculated from weight in kilogrammes divided by height in metres squared, and WHR was calculated as waist measurement divided by hip measurement [22]. Obesity was defined as BMI ≥ 30 kg/m^2^. Central obesity was defined as WHR ≥ 0.85 in women and WHR ≥ 0.90 in men.

Area-based socioeconomic status was measured by the Townsend score, which was derived from census data on housing, employment, social class, and car availability by postcode of residence [23]. A higher Townsend score represents a higher level of deprivation. More detailed information can be found in the UK Biobank online protocol (http://www.ukbiobank.ac.uk).

In the baseline assessment, participants self-reported their physical activity level using the International Physical Activity Questionnaire (IPAQ) [24]. Low physical activity level was defined as < 600 MET-min/week [24].

Dietary information was collected via the Oxford WebQ questionnaire which is based on self-reported 24-h recall. It records the usual consumption of a range of foods and was designed for use in large population studies [25]. Participants were invited on five occasions to complete an online questionnaire between April 2009 and June 2012. For participants who completed more than one questionnaire, we derived the average intake from the questionnaires completed. Total energy intake and energy derived from each macronutrient were calculated, in kilocalories per day, using the information recorded in the 7th edition of McCance and Widdowson’s The Composition of Foods [26]. High total energy intake was defined as > 2,000 kcal/day for women and > 2500 kcal/day for men, in accordance with the NHS guideline (https://www.nhs.uk/live-well/healthy-weight/managing-your-weight/understanding-calories/). The sample size for analysis using these energy intake variables was 95,437.

Since the dietary recall was available in less than half of the UK Biobank participants, this study used a cumulative dietary quality score [27] from the food frequency questionnaire, which was completed by most participants. Twenty-one of the 27 items in the score were deemed to be relevant to the study and therefore included: cooked vegetables, salad/raw vegetables, fresh fruit, dried fruit, oily fish, non-oily fish, processed meat, poultry, beef, lamb, pork, cheese, milk type used, spread type, bread type, cereal intake, cereal type, salt added to food, tea, coffee, and water. We then collapsed beef, pork, and lamb into red meat; oily fish and non-oily fish into total fish; and fresh fruit, dried fruit, salad vegetables, and cooked vegetables, into fruit and vegetables, resulting in 15 items. Six of them have unknown or uncertain associations with health outcomes (such as poultry) or were not available for the full cohort (such as cereal type) and, therefore, were not included in the cumulative dietary quality score. Finally, we included the remaining nine of the 15 food items in the score (processed meat, red meat, total fish, milk, spread type, cereal intake, salt added to food, water, and fruits and vegetables). As food item data were collected using various frequencies of consumption (such as never, less than once a week, or once a week), all food items were dichotomised into meeting or not meeting recommendations using cut-offs derived from the UK and European food-based dietary guidelines (the Eatwell Guide [28] and the Food-Based Dietary Guidelines from the European Food Safety Authority [29]) or median food intake where specific recommendations did not exist [30]. We assigned one point to participants for each healthy category met, defined as processed meat less than once per week [28, 31]; red meat less than once per week [28, 31]; total fish more than twice per week [28]; no consumption of full-cream milk or non-dairy milk [28, 29]; no intake of spread [30]; more than five bowls per week of cereal [30]; no salt added to food [28, 29]; more than six glasses per day of water; and more than five servings per day of fruit and vegetables [28, 29]. Participants’ points were summated to create an unweighted score, with a minimum score of 0 representing the least healthy diet, and a maximum score of 9 representing the healthiest diet. Low diet quality was defined as a diet quality score < 5.

Smoking status was self-reported at baseline and classified as either ever smoker (current or former smoker) or never smoker. Alcohol intake was self-reported as the number of units consumed per week and > 14 units/week was defined as high alcohol intake. Self-reported sleep duration was categorised into abnormal sleep duration (< 7 h/day or > 9 h/day) and normal sleep duration (7–9 h/day) [16].

### Exposures

For this study, we used the updated genetic data (October 2018), which is available on 488,377 participants [32]. Of these, 438,427 samples were genotyped using Affymetrix UK Biobank Axiom Array with 825,927 markers (Santa Clara, CA, USA), and the remaining 49,950 were genotyped using the Affymetrix UK BiLEVE Axiom array with 807,411 markers. These two arrays are extremely similar (sharing more than 95% same content). To maximise homogeneity and BMI-PRS applicability, we exclude participants who did not self-report their ethnicity as white British, and those with missing data on BMI-PRS and WHR-PRS. Further information on the genotyping process is available on the UK Biobank website (http://www.ukbiobank.ac.uk/scientists-3/genetic-data), which includes detailed technical documentation (https://www.nature.com/articles/s41586-018-0579-z).

We used a standard set of sample quality-control procedures, applying statistical tests designed mainly to check for consistency of genotype calling across experimental factors and the indicators of missing rate and heterozygosity to identify poor quality samples, conducting quality control specific to the sex chromosomes using a set of high-quality markers on the X and Y chromosomes [32]. We only used markers present on both the UK BiLEVE and UK Biobank Axiom arrays and excluded those that markers failed to pass the quality control in more than one batch, had a greater than 5% overall missing rate, and had < 0.0001 minor allele frequency (MAF). We removed samples that were identified as outliers for heterozygosity and missing rate [32].

LDpred [33] was used to generate the BMI-PRS [34] and WHR-PRS [19]. LDpred adjusts GWAS summary statistics to account for linkage disequilibrium (LD) between SNPs, creating a single genome-wide score using an infinitesimal model. The raw summary statistics are adjusted using 1000 unrelated UK Biobank participants as the LD reference panel, who were not used in the main analyses. These participants are white British, whose self-reported sex match their genetically determined sex, who do not have purported sex chromosome aneuploidy, and who are not determined by UK Biobank to be outliers for heterozygosity. Scores are then generated using these LD-adjusted summary statistics in those who pass the same genetic quality control as above and were not used in the LD reference panel.

### Statistical analyses

Participant characteristics were firstly compared by BMI-PRS and WHR-PRS categories. The weighted PRS scores were transformed into *z* scores and categorised as PRS <  − 1 (i.e. more than 1 standard deviation (SD) below the mean), − 1 < PRS < 0, 0 < PRS < 1, and PRS > 1. All the lifestyle factors were classified as binary variables in the pre-specified deleterious direction. The sociodemographic and lifestyle characteristics of the PRS categories were summarised using frequencies and percentages and compared using chi-square tests.

The first set of analysis focuses on mediation. Logistic regression was used to investigate whether lifestyle factors mediated the associations between PRS and obesity. In this analysis, BMI-PRS and WHR-PRS were the exposures, and the outcomes were obesity (BMI ≥ 30 kg/m^2^) and central obesity (WHR ≥ 0.9 for men and WHR ≥ 0.85 for women), respectively. We tested for statistical interactions between sex and BMI-PRS and WHR-PRS. Where the interactions were significant, the models were run stratified by sex. The models were adjusted for potential confounders (age, sex, and deprivation), the 10 principal genetic components (PGC) (to correct for population stratification), and the genotyping chip used. Each of the lifestyle factors was then added sequentially.

The second analysis focuses on interaction. Stratified logistic regression models were then used to test for interactions between PRS and lifestyle factors. Separate models were run for each lifestyle factor. Interaction terms were included to investigate whether individual lifestyle factors interacted with obesity-related PRS (low PRS defined as PRS < 0; high PRS defined as ≥ 0) on the multiplicative scale. The additive interaction metric, relative excess risk due to interaction (RERI), was also derived [35]. The dichotomisation of PRS is required for calculating RERI and is only used for this analysis.

Finally, a 4-way decomposition was used to quantify how much of the total association between BMI-PRS/WHR-PRS and obesity and central obesity could be attributed to mediation, additive interaction, or neither [36]. The association between PRS and outcome was estimated as an odds ratio (OR) in the logistic regression model. This method further decomposes the OR (‘total effect’) into: OR via interaction with lifestyle (‘effect due to interaction’), OR via mediation through lifestyle (‘mediated effect’), and OR not via lifestyle (‘direct effect’). Because the logistic regression model operates on the logistic scale, it could be interpreted as total effect = effect due to interaction * effect due to mediation * effect not due to lifestyle. The results were presented as the overall proportion of excess prevalence attributable to additive interaction ([effect due to interaction − 1]/[total effect − 1]) and mediation ([mediated effect − 1]/[total effect − 1]). The total lifestyle in 4-way decomposition analyses represents the mediation/modification role of when combining all lifestyles together (PAL, diet quality, etc.), and adjusting age, sex, deprivation, genetic principal components and chip. In a sensitivity analysis, all lifestyle factors were also adjusted mutually for a conservative estimate. Because there is a slight difference in BMI-PRS by sex, we conducted a sensitivity to adjust for BMI-PRS*sex interaction as a covariate in the final 4-way decomposition analysis. All statistical analyses were conducted using R, with the cmest function from the CMAverse package and two-sided P < 0.05 was considered statistically significant.

## Results

### Study population characteristics

UK Biobank is a prospective general population cohort of approximately *N* = 502,000. The study population here comprised ultimately 201,466 participants with complete data, with a mean age of 58 years, of whom 101,278 (50.3%) were female. Participants with higher BMI-PRS and WHR-PRS scores were less deprived and more likely to be male (Table 1). Overall, 33,889 (16.8%) participants had low levels of physical activity (< 600 MET-min/week), 58,839 (29.2%) had poor quality diets (diet quality score < 5), 48,237 (23.9%) had abnormal sleep duration (> 9 or < 7 h/day), 96,225 (47.8%) had high alcohol consumption (> 14 unit/week), and 92,084 (45.7%) were ever smokers. Of the 95,457 participants with dietary energy information, 36,940 (38.7%) had high energy intake (> 2000 kcal/day for women and > 2500 kcal/day for men).

**Table 1:** Participant characteristics by BMI and WHR-PRS categories (measured by *z*-score and presented by each SD)

Phenotype	BMI-PRS < − 1 *N* = 32,760	BMI-PRS − 1 to 0 *N* = 68,679	BMI-PRS 0 to 1 *N* = 68,809	BMI-PRS > 1 *N* = 31,198	*P* value	WHR-PRS < − 1 *N* = 32,692	WHR-PRS − 1 to 0 *N* = 69,153	WHR-PRS 0 to 1 *N* = 68,108	WHR-PRS > 1 *N* = 31,493	*P* value
Age, years, median (IQR)	58 (50, 63)	58 (50, 63)	58 (50, 63)	57 (50, 63)	< 0.001	58 (50, 63)	58 (50, 63)	58 (50, 63)	57 (50, 63)	< 0.001
Deprivation index, median (IQR)	− 2.52 (− 3.87, − 0.34)	− 2.50 (− 3.83, − 0.30)	− 2.46 (− 3.80, − 0.21)	− 2.40 (− 3.76, − 0.11)	0.992	− 2.52 (− 3.86, − 0.38)	− 2.53 (− 3.83, − 0.33)	− 2.45 (− 3.79, − 0.18)	− 2.37 (− 3.75, − 0.05)	0.994
Sex										
Female	16,627 (51%)	34,657 (50%)	34,630 (50%)	15,364 (49%)	< 0.001	16,500 (50%)	34,811 (50%)	34,200 (50%)	15,767 (50%)	0.736
Male	16,133 (49%)	34,022 (50%)	34,179 (50%)	15,834 (51%)		16,192 (50%)	34,342 (50%)	33,908 (50%)	15,726 (50%)	
Low physical activity level	5431 (17%)	11,328 (16%)	11,639 (17%)	5491 (18%)	< 0.001	5319 (16%)	11,447 (17%)	11,629 (17%)	5494 (17%)	< 0.001
Low diet quality	9635 (29%)	20,053 (29%)	19,960 (29%)	9191 (29%)	0.400	9008 (28%)	19,950 (29%)	20,270 (30%)	9611 (31%)	< 0.001
High dietary energy intake	6503 (40%)	12,815 (39%)	12,353 (38%)	5269 (37%)	< 0.001	6516 (40%)	12,947 (39%)	12,145 (38%)	5332 (38%)	0.024
Abnormal sleep duration	7493 (23%)	16,090 (23%)	16,672 (24%)	7982 (26%)	< 0.001	7293 (22%)	16,270 (24%)	16,685 (24%)	7989 (25%)	< 0.001
High alcohol consumption	15,696 (48%)	32,956 (48%)	32,858 (48%)	14,715 (47%)	0.107	15,484 (47%)	32,752 (47%)	32,742 (48%)	15,247 (48%)	0.002
Ever smoker	14,473 (44%)	31,054 (45%)	31,741 (46%)	14,816 (47%)	< 0.001	14,339 (44%)	31,124 (45%)	31,708 (47%)	14,913 (47%)	< 0.001

*Abbreviations*: *BMI* Body mass index, *WHR* Waist-hip ratio, *PRS* Polygenic risk scores

Numbers presented are the median (*IQR*, interquartile range) for continuous variables and the number (percent) for categorical variables (*N* = 201,466). Range for the BMI-PRS and WHR-PRS are as follows: Q1, <  − 1 s.d.; Q2, − 1 to 0 s.d.; Q3, 0 to 1 s.d.; Q4, > 1 s.d

### Mediation roles of lifestyle factors

There was a significant interaction between sex and WHR-PRS in relation to phenotypic central obesity (*P* < 0.001), but there was no interaction between sex and BMI-PRS in relation to phenotypic obesity (*P* = 0.446). Therefore, all subsequent analysis using WHR-PRS is sex-stratified.

BMI-PRS and WHR-PRS were associated with higher odds of low physical activity level, abnormal sleep duration, and ever smoking, whilst WHR-PRS was associated with poor quality diet. Conversely, BMI-PRS and WHR-PRS were associated with lower odds of high dietary energy intake in both sexes and high alcohol intake in women (Additional file 1: Fig. S1 and S2).

All unhealthy lifestyle habits were significantly associated with higher odds of obesity and central obesity both in men and women, apart from high alcohol consumption which was significantly associated with lower odds of obesity. High dietary energy intake which was not significantly associated with the prevalence of central obesity in men (Additional file 1: Fig. S3 and S4).

Both BMI-PRS and WHR-PRS were significantly associated with phenotypic obesity and central obesity (Additional file 1: Fig. S5 and S6). Following adjustment for lifestyle risk factors, the effect sizes were not attenuated and the associations between both PRS scores and phenotypic obesity and central obesity remained statistically significant.

### Interactions between PRS and lifestyle

**Fig. 2:**
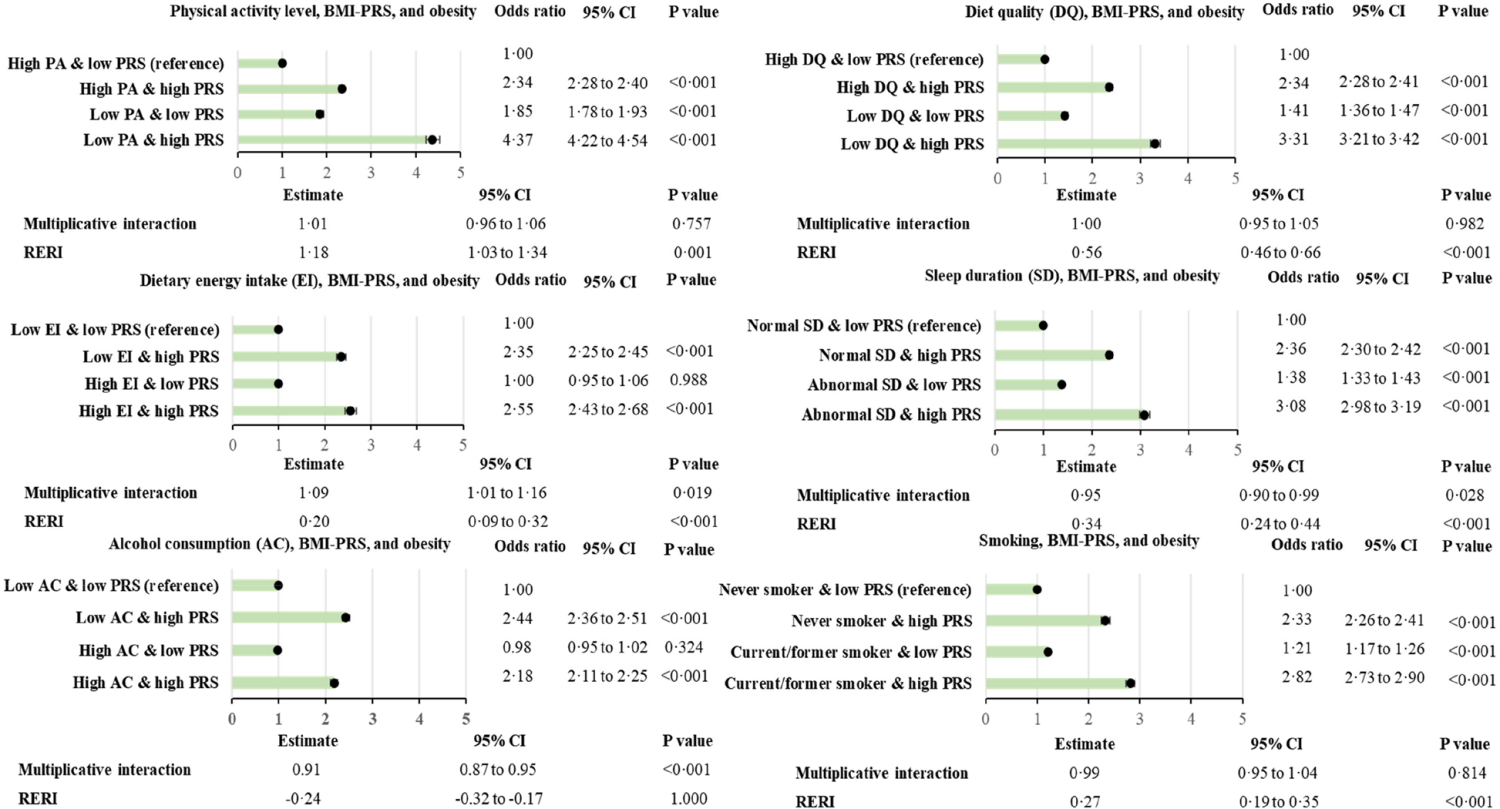
Combined association of BMI-PRS and lifestyle risk factors with obesity. Adjusted for age, sex, deprivation index, 10 principal genetic components, and chip

**Fig. 3:**
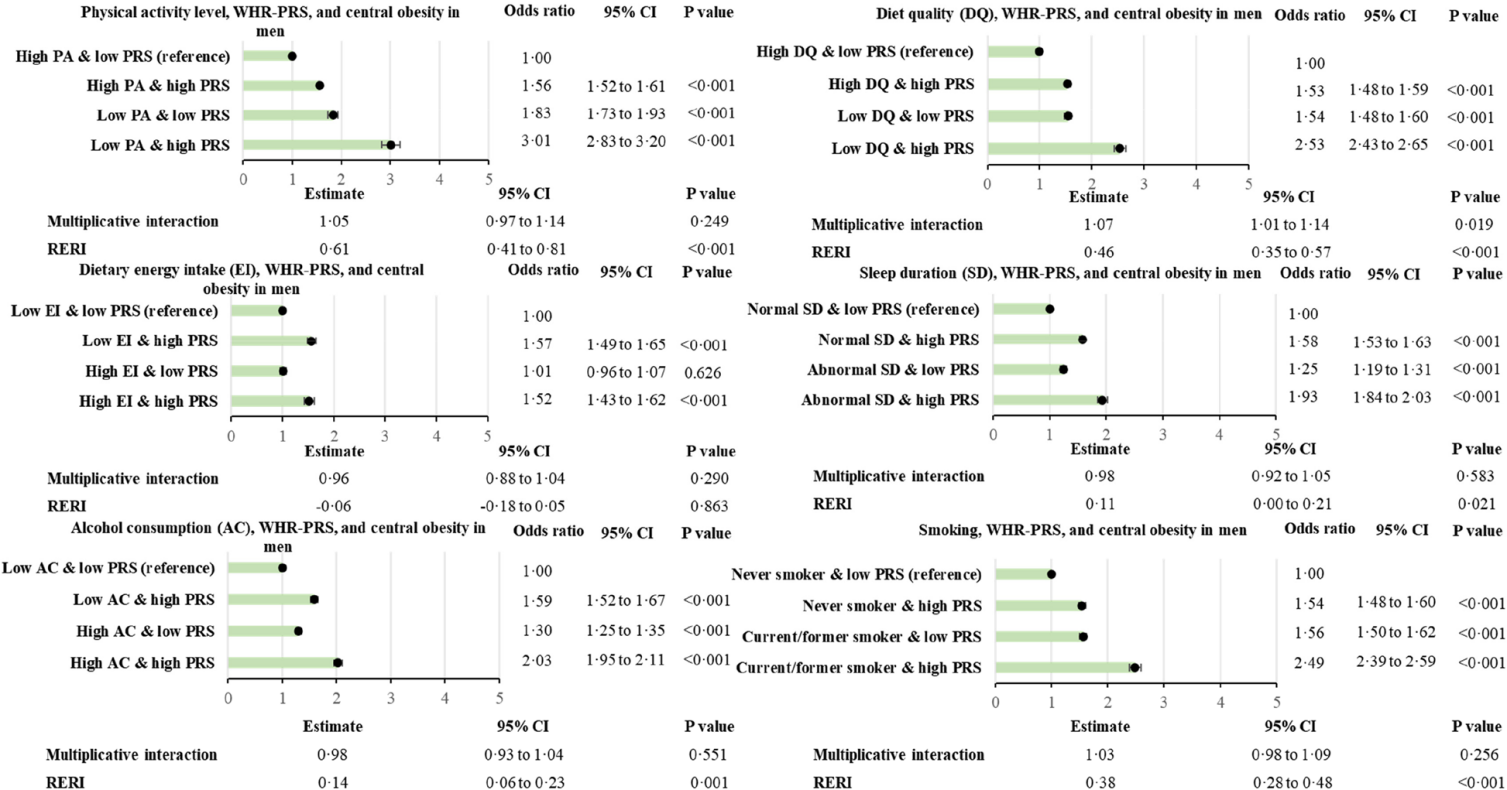
Combined association of WHR-PRS and lifestyle risk factors with central obesity in men. Adjusted for age, deprivation index, 10 principal genetic components, and chip

**Fig. 4:**
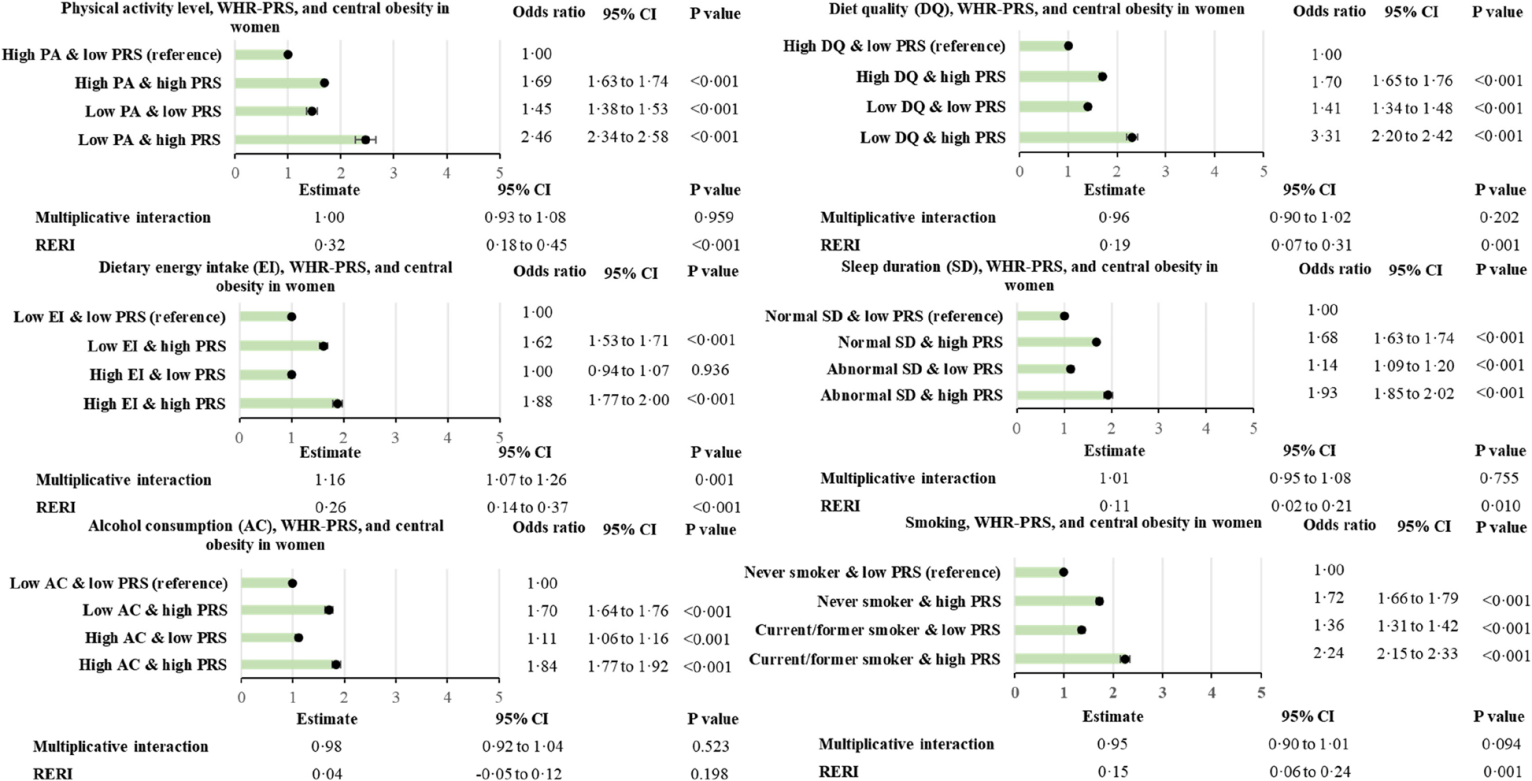
Combined association of WHR-PRS and lifestyle risk factors with central obesity in women. Adjusted for age, deprivation index, 10 principal genetic components, and chip

Figure 2 presents the association between obesity and the different combinations of BMI-PRS score and lifestyle factors. Among people with a low genetic predisposition to obesity, high energy intake and high alcohol consumption were not significantly associated with increased prevalence of obesity (Fig. 2). For all of the lifestyle factors, the additive interactions (RERI) were all significant except for high alcohol intake. Results are similar for WHR-PRS and central obesity (Figs. 3 and 4). All RERIs were significant except for energy intake in men and alcohol intake in women. However, in men and women with a low genetic predisposition category, high energy intake was not significantly associated with an increased prevalence of phenotypic central obesity (Figs. 3 and 4). The interactions were all significantly associated with RERIs except for the interaction between WHR-PRS in females and alcohol intake, and the interaction between WHR-PRS in males and energy intake. In contrast to additive interactions, multiplicative interactions did not reach statistical significance, except for BMI-PRS and energy intake, alcohol consumption, and sleep duration, WHR-PRS and diet quality in men, and WHR-PRS and total energy intake in women.

### Combined interaction and mediation effects

**Fig. 5:**
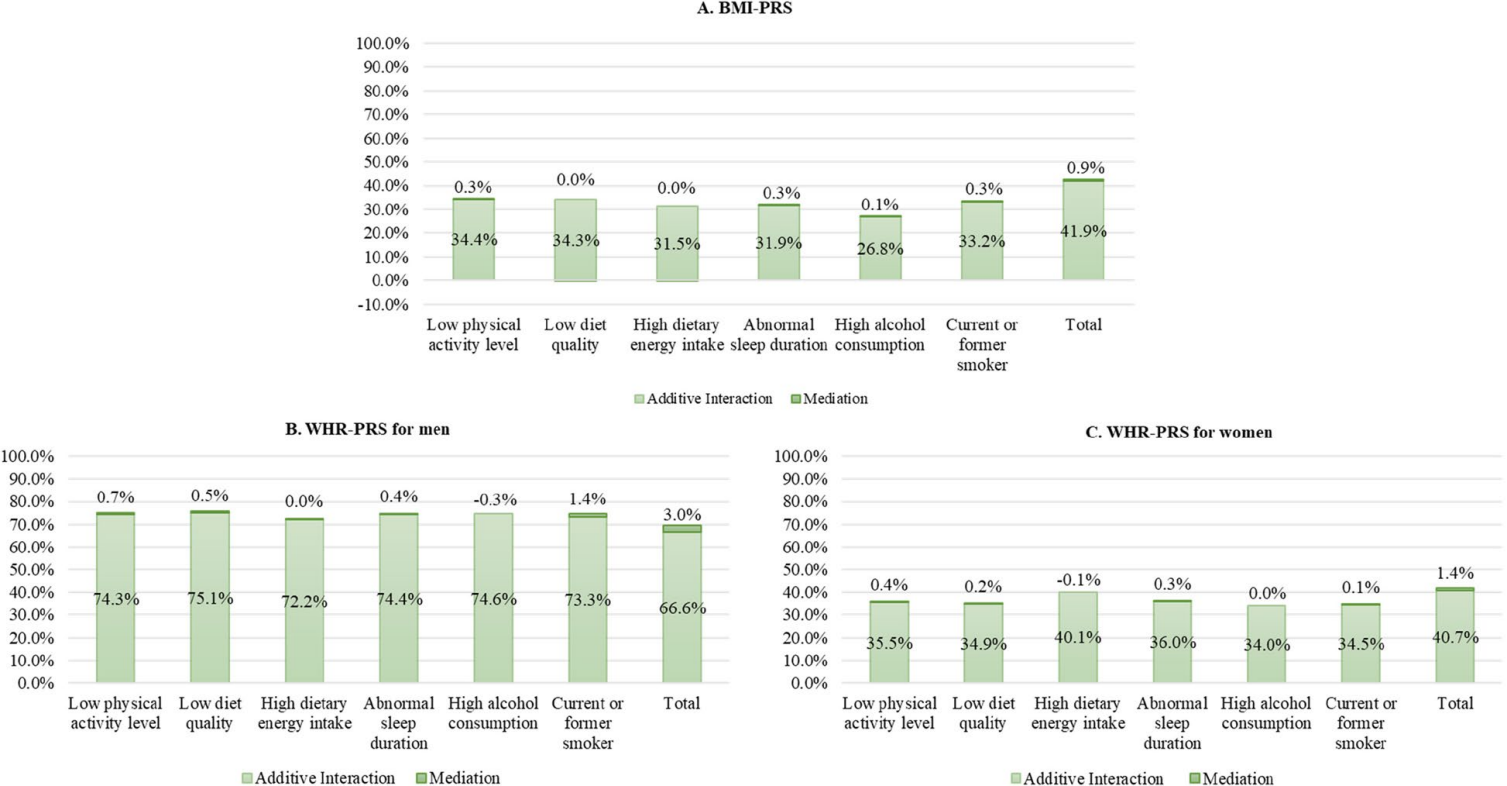
The proportion of excess risk due to BMI/WHR-PRS is attributable to the interaction and mediation of lifestyle risk. Estimated from 4-way decomposition analysis. Adjusted for age, deprivation index, 10 principal genetic components, and chip

Figure 5 summarises the overall contribution of each lifestyle risk factor, through interaction and mediation, to the association between genetic predisposition and phenotypic obesity and central obesity, and the contribution of lifestyle as a whole. All interactions were statistically significant (*p* < 0.001).

Overall, 42.8% of the association between BMI-PRS and phenotypic obesity could be explained by overall lifestyle; 0.9% was mediated via lifestyle and 41.9% was attributable to effect modification by lifestyle (Fig. 5 panel a). Among women, 42.1% of the association between WHR-PRS and phenotypic central obesity was explained by lifestyle; 1.4% was mediated by lifestyle and 40.7% was attributable to interactions with lifestyle (Fig. 5 panel c). The contribution of lifestyle was greatest in relation to central obesity in men, with 69.6% of the association between WHR-PRS and phenotypic central obesity explained by lifestyle; 3.0% mediated by lifestyle and 66.6% attributable to interactions with lifestyle (Fig. 5 panel b).

The findings were largely consistent with slight changes in estimates in the 4-way decomposition analysis with the additional adjustment of BMI-PRS * sex interaction (Additional file 1: Fig. S7).

## Discussion

Our findings in white participants of the UK Biobank suggest that genetic predisposition to obesity and central obesity does not inevitably lead to phenotypic obesity and central obesity. Within the cohort studied, almost half of the association between genetic predisposition to obesity and phenotypic central obesity could be explained by lifestyles that increased the likelihood of genetic predisposition being realised. If these findings were proven causal, which we were not able to establish in this study, almost half of the effect of their genetic predisposition was potentially avoidable by lifestyle modification. In men, the contribution of lifestyle to phenotypic central obesity was even higher, with lifestyle accounting for 70% of the likelihood of genetic predisposition being realised.

Individuals with a higher genetic predisposition to obesity and/or central obesity were more likely to have low physical activity levels, abnormal sleep duration and poor-quality diets. This suggests that individuals with high genetic predisposition may be more likely to benefit most from health promotion interventions focused on these lifestyle factors. Within the population studied, people with higher genetic predisposition to obesity and central obesity were also healthier in some regards; specifically, lower energy and alcohol intake. It is possible that some of the differences in lifestyle reflected vertical pleiotropy; the same genes predisposing to poor lifestyle and thereby to obesity and central obesity. However, our findings suggested that very little of the association between genetic predisposition and phenotypic obesity and central obesity was mediated via lifestyle. It is also plausible that some of the differences in lifestyle reflect obesity-related lifestyle choices whereby people who know they are prone to weight gain choose to restrict their energy intake either overall or via reduced alcohol consumption or choose to smoke because of the negative association between smoking and weight [37].

Our findings for interaction analyses suggest that, in general, both genetic predisposition and an unhealthy lifestyle (specifically lack of physical activity, abnormal sleep duration and poor-quality diet) increase the prevalence of obesity and central obesity, in addition to the above lifestyle factors, smoking status and alcohol consumption also have a similarly large effect on central obesity both in male and female. And they both lead to an even higher prevalence. However, whilst high genetic predisposition increases prevalence irrespective of lifestyle, the adverse effect of consuming excess calories is restricted to those who have a higher genetic predisposition. Additionally, it is not meaningful to study the role of a single lifestyle factor with obesity solely when the interaction is significant and apparent, and the magnitude of the role of the factor must be studied by considering different levels of other lifestyle factors [35].

Our findings in relation to BMI are consistent with previous prospective studies suggesting that higher BMI-PRS correlates with lower physical activity levels [38, 39], and the association between BMI-PRS and obesity was moderated by diet quality [40–42], alcohol intake [43], sleep duration [16], and physical activity levels [44, 45]. Fewer studies have investigated whether lifestyle moderates the association between WHR-PRS and central obesity. Only in a study of 68,317 people of European ancestry, was there a small but statistically significant interaction between dietary score and BMI-adjusted WHR in relation to WHR-PRS [42].

Previous studies on mediation have focused on physical activity and diet, specifically whether genetic predisposition is mediated via individual differences including eating behaviour and appetite. Both emotional eating and eating disorders have been shown to mediate the relationship between BMI-PRS and obesity [46], and individuals at a higher genetic prevalence of obesity tended to be more habitually and situationally disinhibited when eating and had a greater tendency to feel hungry in response to the environment, resulting in a greater prevalence of obesity [47]. The mediating role of physical activity has been investigated specifically in relation to the fat mass and obesity-associated (*FTO*) genotype, with studies observing that both sedentary time and physical activity level mediated the association between *FTO* genotype and BMI [48, 49]. Chuang et al. demonstrated in 697 participants that a substantial amount of the association between *FTO* and adiposity was mediated by personality aspects (e.g. excitement-seeking), high energy consumption, and aspects of brain function (as indexed by functional magnetic resonance imaging) [50].

The strengths of this study firstly, investigation of two different measures of adiposity in the same large, population cohort: BMI as a measure of overall obesity and WHR as a measure of central obesity. Secondly, having observed a significant interaction with sex, sex-stratified analyses were conducted for all analyses involving WHR-PRS. Finally, we were able to investigate a wide range of lifestyle factors; whether they were associated with obesity and central obesity independently of each other, whether they mediated the association between genetic predisposition to obesity and central obesity, and whether they modified these associations.

One of the limitations of the study was that the UK Biobank is not representative of the UK population in terms of the demographic and lifestyle characteristics of participants [51]. Therefore, whilst effect sizes can be generalised, prevalence and estimates derived from prevalence should not be automatically generalised. Since the lifestyle of UK participants is generally healthier than the general population, it is likely that the attributable percentages may be larger in the general population than those reported here. Within the UK Biobank, European participants were the only ethnic group sufficiently powered to study genetic interactions [52] and, owing to different genetic susceptibilities and lifestyles, our findings should not be generalised to other ethnic groups, so the role of lifestyle in these groups remains unknown pending further research [53]. Secondly, whilst we adjusted for known, measured confounders such as age, sex and socioeconomic status, residual confounding is possible in any observational study. Lifestyle factors were self-reported and potentially subject to measurement error and reporting bias [54], and lifestyle factors and adiposity were measured at a single time-point but may vary over time. Future studies are required to corroborate our findings in more diverse populations with different lifestyle profiles. Whilst we investigated a range of lifestyle factors but could not identify weight type when it increased because of increment of lean body mass, future studies should investigate additional factors, such as psychological traits and appetite [55], and identify specific different weight types, to broaden our understanding of gene-lifestyle interactions and mediation. Finally, given that our study used cross-sectional data, the findings could not prove causality between the mediators and obesity, or on the development of obesity [56]. It is particularly difficult to disentangle the relationship between lean mass, obesity, and energy intake as obesity development also increases lean mass which could require higher energy intake. The association between alcohol and obesity could also be subject to various biases as in other epidemiologic studies [57].

## Conclusions

In summary, the study shows that the extent to which genetic predisposition results in phenotypic obesity and central obesity is primarily, and significantly modified by lifestyle. If the causal assumption in this study holds, people who were genetically predisposed to obesity and central obesity were particularly suspectable to the effect of lifestyle.

### Supplementary Information

Supplementary Material 1.

## Data Availability

Data extracted from this study and data used for all analysis are from the UK Biobank database which were reported in this paper. No additional data are available. More information about the UK Biobank can be found online (http://www.ukbiobank.ac.uk).

## Abbreviations

AC: Alcohol consumption
BMI: Body mass index
CI: Confidence interval
DQ: Diet quality
EI: Energy intake
FTO: Fat mass and obesity-associated
IQR: Interquartile range
LD: Linkage disequilibrium
OR: Odds ratio
PA: Physical activity level
PGC: Principal genetic components
PRS: Polygenic risk scores
RERI: Relative excess risk due to interaction
SD: Sleep duration
SD: Standard deviation
WHR: Waist-hip ratio
