# Supplementary material for: How do lifestyle factors modify the association between genetic predisposition and obesity-related phenotypes? A 4-way decomposition analysis using UK Biobank
Source: BMC Med. 2024 Jun 10;22:230. doi: 10.1186/s12916-024-03436-6 (PMC11163778; doi:10.1186/s12916-024-03436-6)
Supplement: Supplementary file 1 — Supplementary Material 1. [file 12916_2024_3436_MOESM1_ESM.pdf]

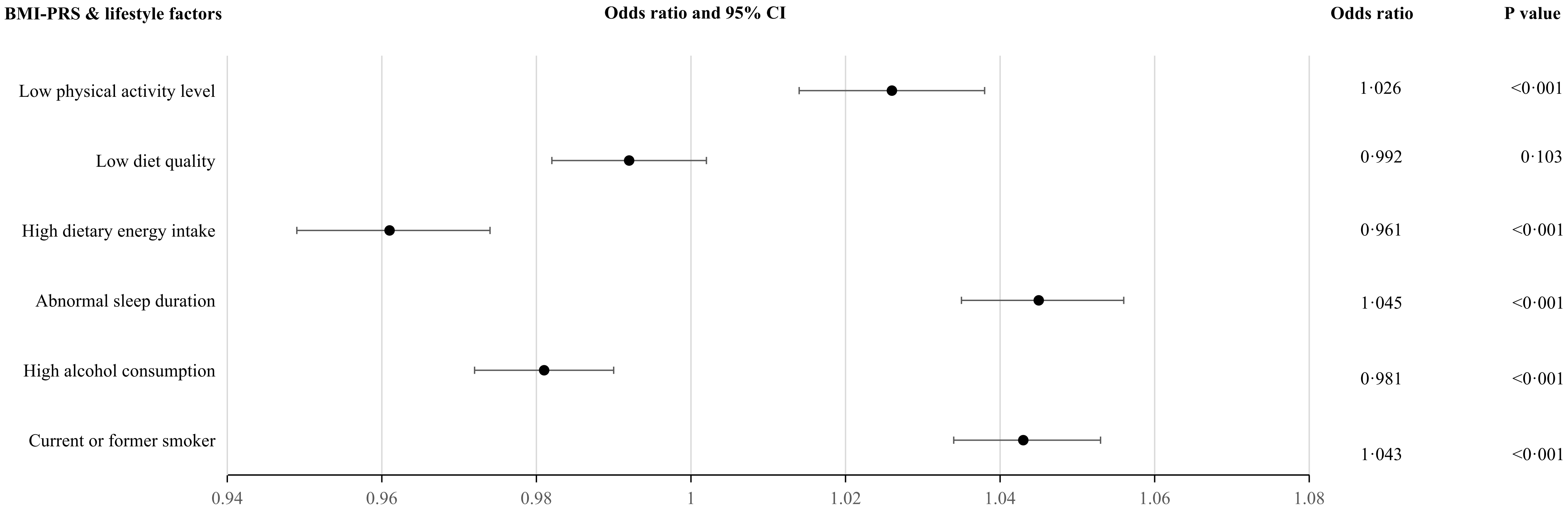

Fig S1. Association between BMI-PRS and lifestyle factors. Adjusted for age, sex, deprivation index, 10 principal genetic components, and chip.

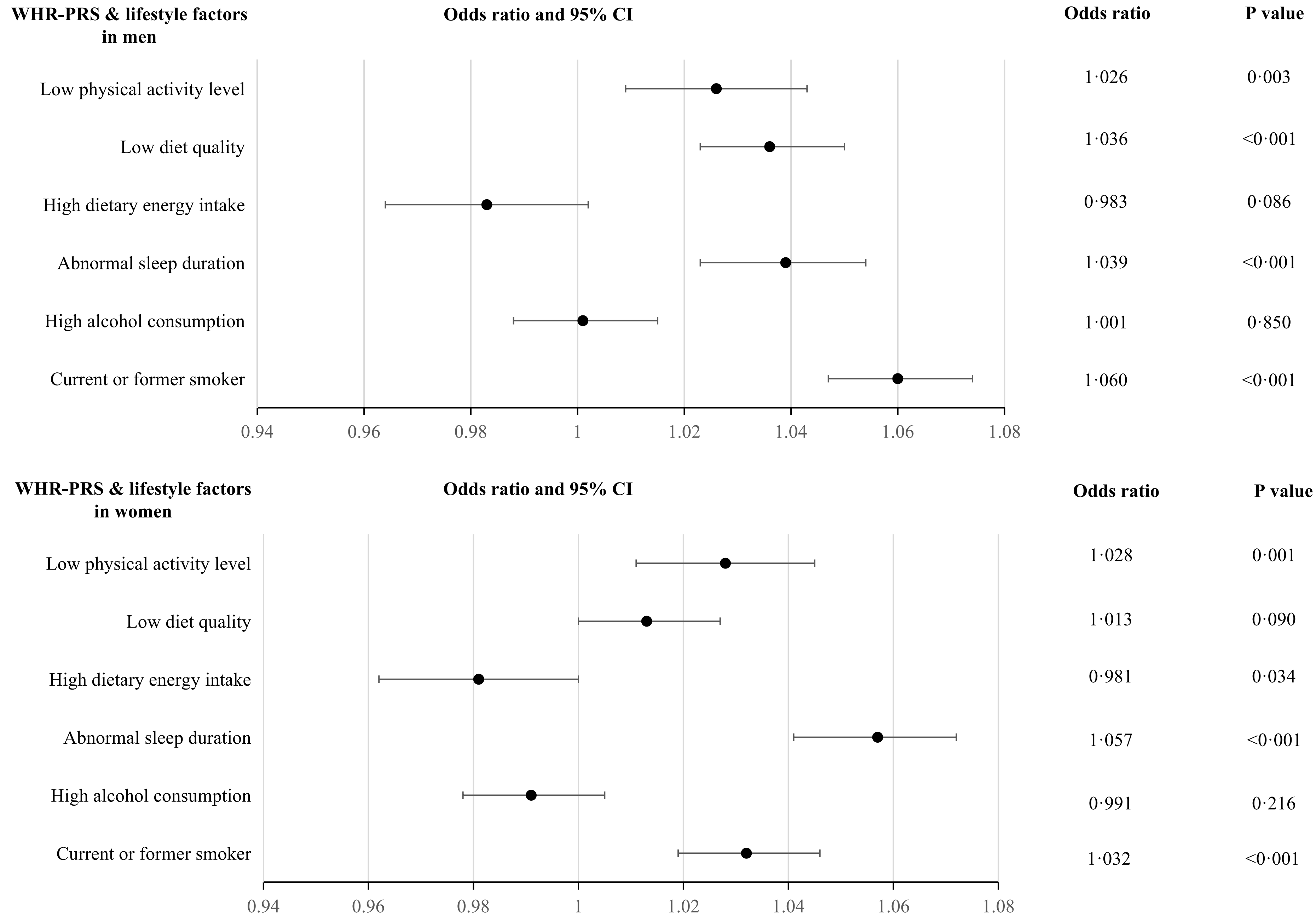

Fig S2. Association between WHR-PRS & lifestyle factors in men and women. Adjusted for age, deprivation index, 10 principal genetic components, and chip.

Lifestyle factor & obesity	Odds ratio	P value
Low physical activity level	1·840	<0·001
Low diet quality	1·394	<0·001
High dietary energy intake	1·038	<0·001
Abnormal sleep duration	1·341	<0·001
High alcohol consumption	0·923	<0·001
Current or former smoker	1·219	<0·001

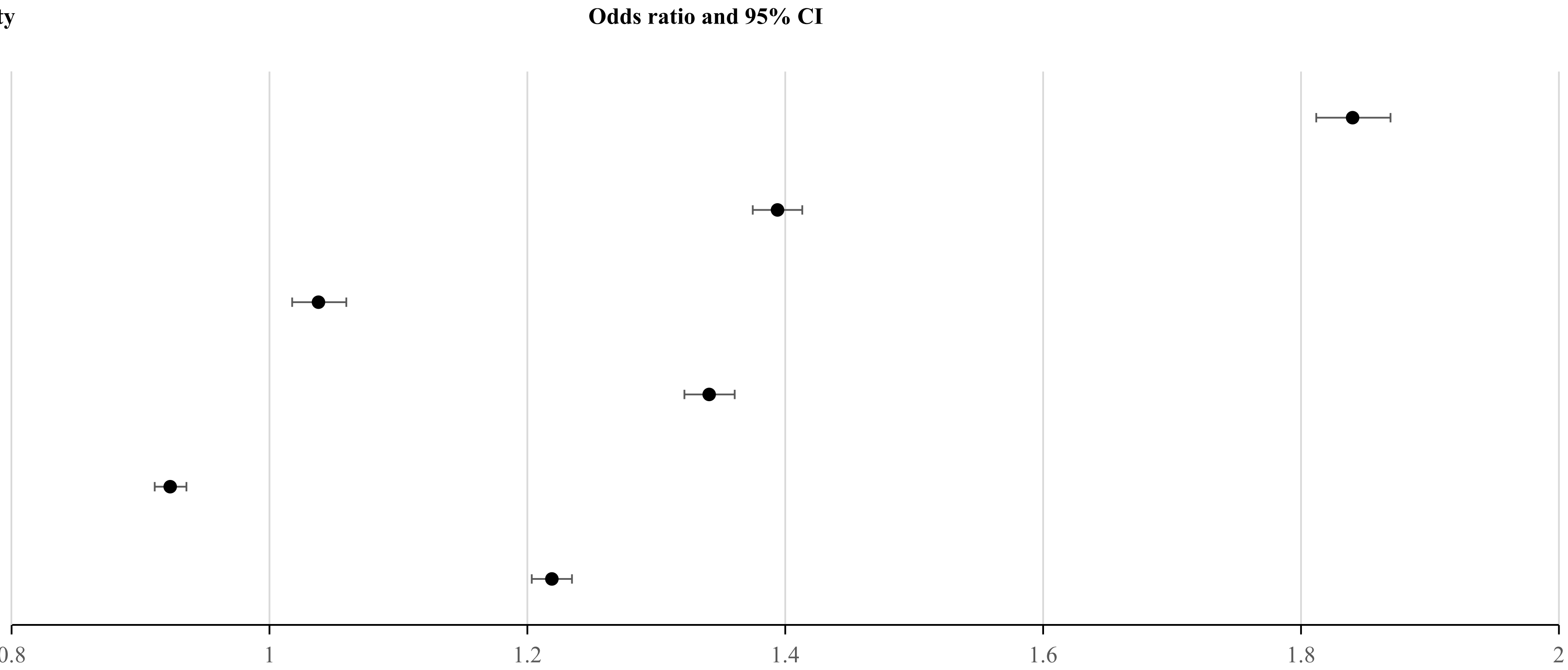

Fig S3. Association between lifestyle factors & obesity. Adjusted for age, sex, deprivation index, 10 principal genetic components, and chip.

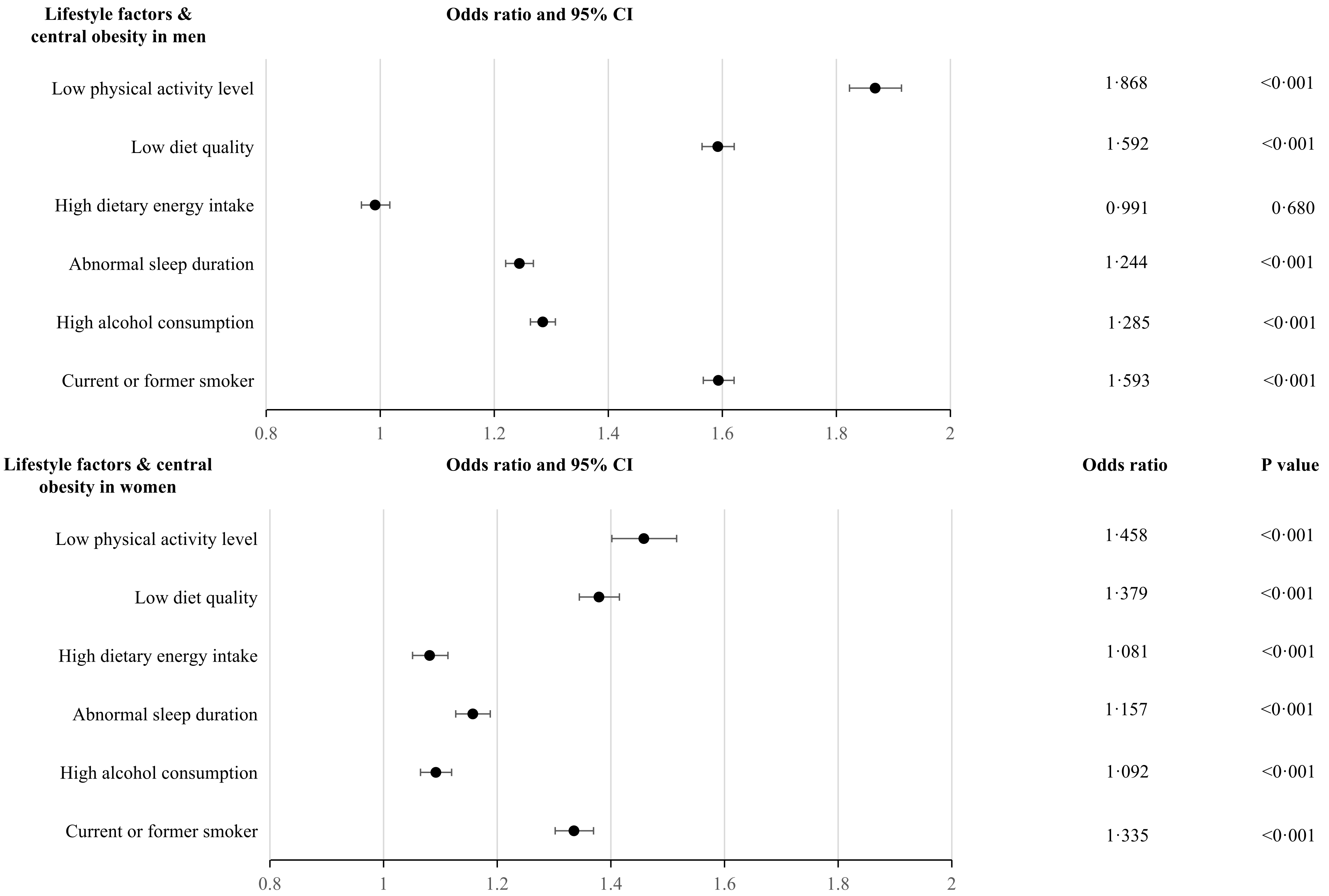

Fig S4. Association between lifestyle factors & central obesity in men and women. Adjusted for age, deprivation index, 10 principal genetic components, and chip.

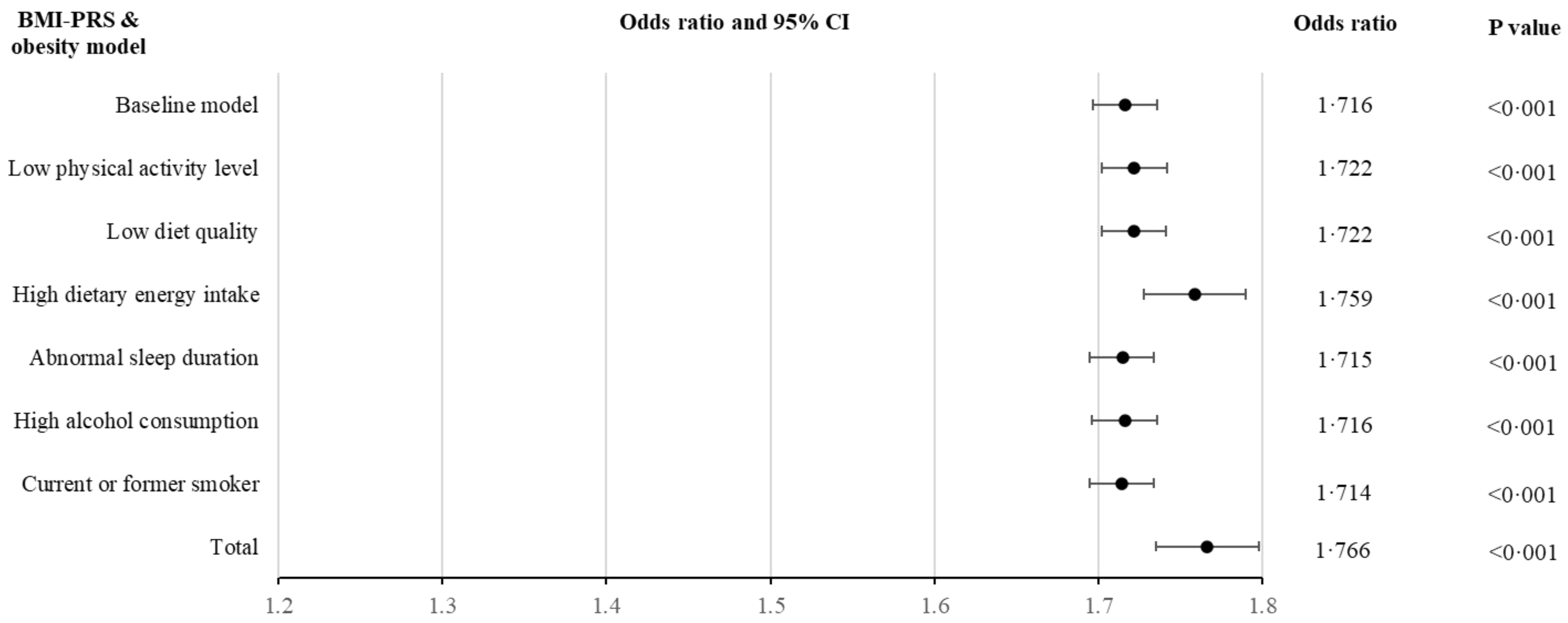

Fig S5. Association between BMI-PRS & obesity by adjustment models. The baseline model adjusted for age, sex, deprivation index, 10 principal genetic components, and chip.

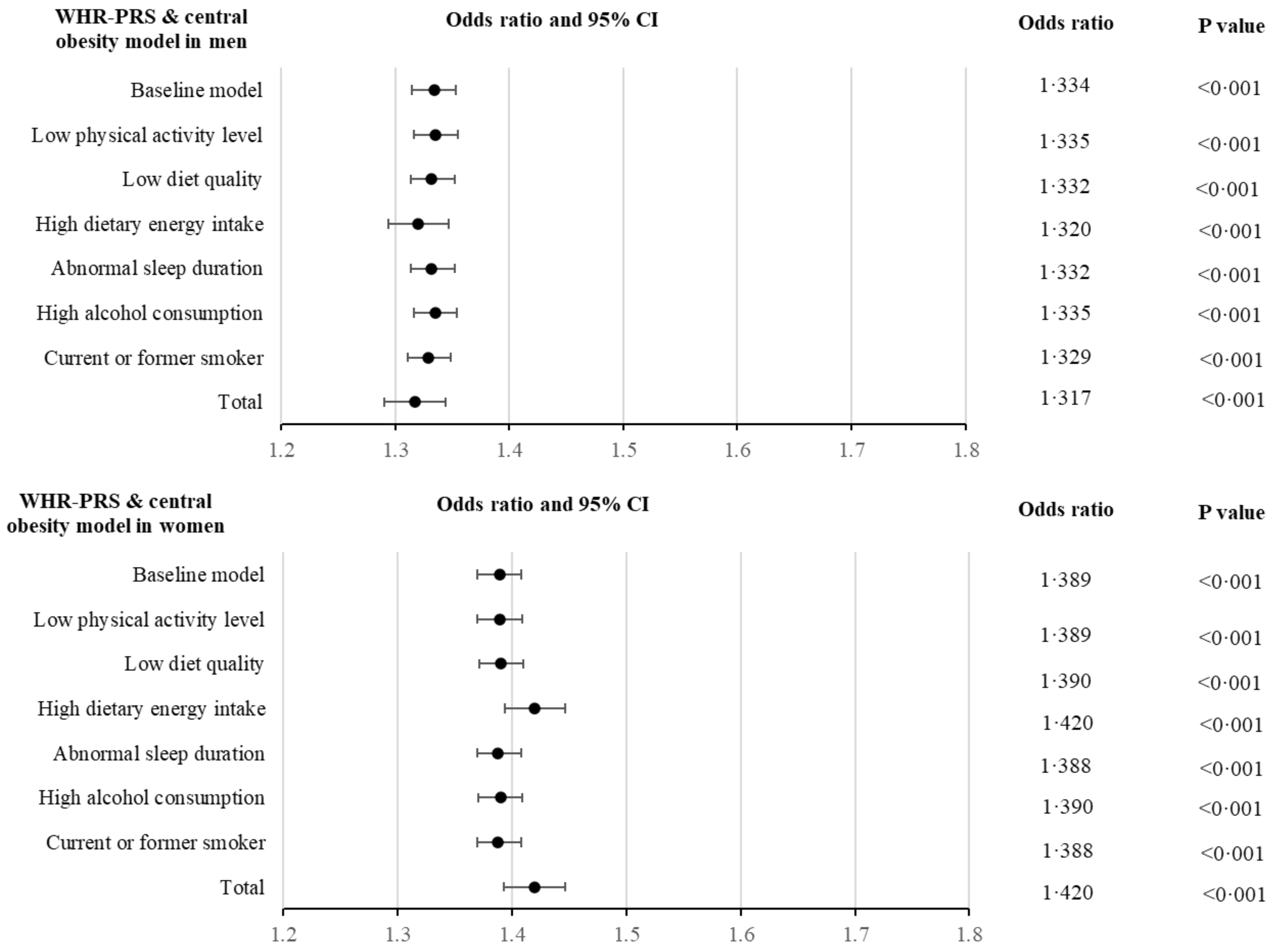

Fig S6. Association between WHR-PRS & central obesity by adjustment models in men and women. The baseline model adjusted for age, deprivation index, 10 principal genetic components, and chip.

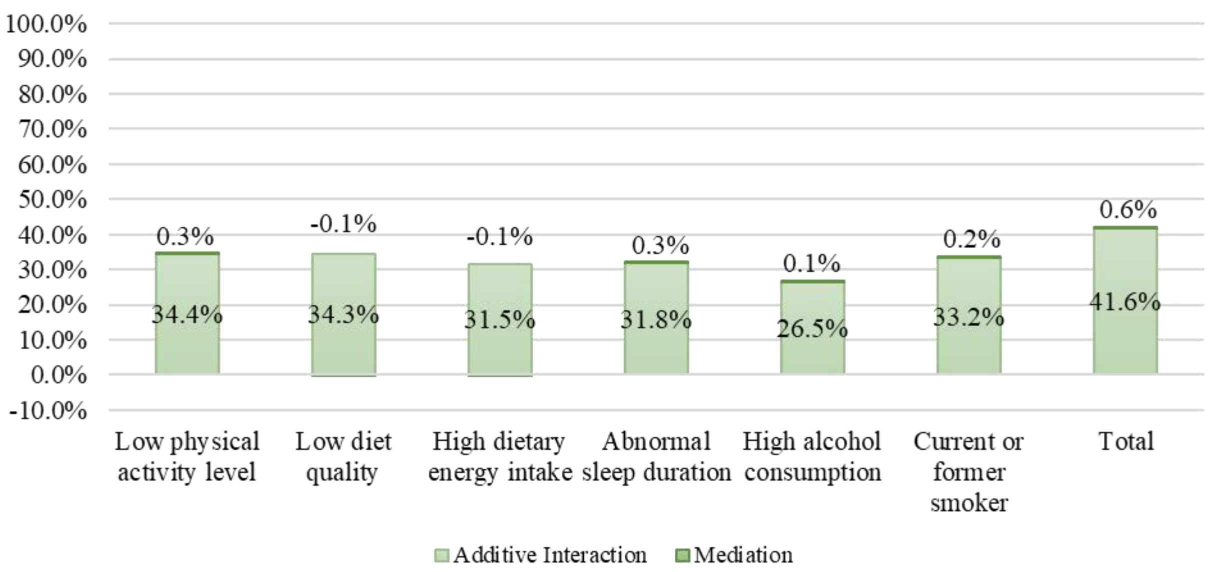

Figure S7. The proportion of excess risk due to BMI-PRS is attributable to the interaction and mediation of lifestyle risk. Estimated from 4-way decomposition analysis. Adjusted for age, deprivation index, 10 genetic principal components, BMI-PRS*sex, and chip.
